# Gastric inhibitory polypeptide receptor antagonism suppresses intramuscular adipose tissue accumulation and ameliorates sarcopenia

**DOI:** 10.1002/jcsm.13346

**Published:** 2023-10-27

**Authors:** Yuya Takahashi, Hiroki Fujita, Yusuke Seino, Satoko Hattori, Shihomi Hidaka, Tsuyoshi Miyakawa, Atsushi Suzuki, Hironori Waki, Daisuke Yabe, Yutaka Seino, Yuichiro Yamada

**Affiliations:** ^1^ Department of Metabolism and Endocrinology Akita University Graduate School of Medicine Akita Japan; ^2^ Department of Endocrinology, Diabetes and Metabolism Fujita Health University Toyoake Japan; ^3^ Yutaka Seino Distinguished Center for Diabetes Research Kansai Electric Power Medical Research Institute Kyoto Japan; ^4^ Division of Systems Medical Science, Center for Medical Science Fujita Health University Toyoake Japan; ^5^ Department of Diabetes, Endocrinology and Metabolism/Department of Rheumatology and Clinical Immunology Gifu University Graduate School of Medicine Gifu Japan; ^6^ Center for One Medicine Innovative Translational Research Gifu University Gifu Japan; ^7^ Center for Diabetes, Endocrinology and Metabolism Kansai Electric Power Hospital Osaka Japan

**Keywords:** aging, fibro‐adipogenic progenitors, GIP receptor, intramuscular adipose tissue, sarcopenia

## Abstract

**Background:**

Intramuscular adipose tissue (IMAT) formation derived from muscle fibro‐adipogenic progenitors (FAPs) has been recognized as a pathological feature of sarcopenia. This study aimed to explore whether genetic and pharmacological gastric inhibitory polypeptide (GIP) receptor antagonism suppresses IMAT accumulation and ameliorates sarcopenia in mice.

**Methods:**

Whole body composition, grip strength, skeletal muscle weight, tibialis anterior (TA) muscle fibre cross‐sectional area (CSA) and TA muscle IMAT area were measured in young and aged male C57BL/6 strain GIP receptor (*Gipr*)‐knockout (*Gipr* ^−/−^) and wild‐type (*Gipr* ^+/+^) mice. FAPs isolated from lower limb muscles of 12‐week‐old *Gipr* ^+/+^ mice were cultured with GIP, and their differentiation into mature adipocytes was examined. Furthermore, TA muscle IMAT area and fibre CSA were measured in untreated *Gipr* ^−/−^ mice and GIP receptor antagonist‐treated *Gipr* ^+/+^ mice after glycerol injection into the TA muscles.

**Results:**

Body composition analysis revealed that 104‐week‐old *Gipr* ^−/−^ mice had a greater proportion of lean tissue mass (73.7 ± 1.2% vs. 66.5 ± 2.7%, *P* < 0.05 vs. 104‐week‐old *Gipr* ^+/+^ mice) and less adipose tissue mass (13.1 ± 1.3% vs. 19.4 ± 2.6%, *P* < 0.05 vs. 104‐week‐old *Gipr* ^+/+^ mice). Eighty‐four‐week‐old *Gipr* ^−/−^ mice exhibited increases in grip strength (*P* < 0.05), weights of TA (*P* < 0.05), soleus (*P* < 0.01), gastrocnemius (*P* < 0.05) and quadriceps femoris (*P* < 0.01) muscles, and average TA muscle fibre CSA (*P* < 0.05) along with a reduction in TA muscle IMAT area assessed by the number of perilipin‐positive cells (*P* < 0.0001) compared with 84‐week‐old *Gipr* ^+/+^ mice. Oil Red O staining analysis revealed 1.6‐ and 1.7‐fold increased adipogenesis in muscle FAPs cultured with 10 and 100 nM of GIP (*P* < 0.01 and *P* < 0.001 vs. 0 nM of GIP, respectively). Furthermore, both untreated *Gipr* ^−/−^ mice and GIP receptor antagonist‐treated *Gipr* ^+/+^ mice for 14 days after glycerol injection into the TA muscles at 12 weeks of age showed reduced TA muscle IMAT area (1.39 ± 0.38% and 2.65 ± 0.36% vs. 6.54 ± 1.30%, *P* < 0.001 and *P* < 0.01 vs. untreated *Gipr* ^+/+^ mice, respectively) and increased average TA muscle fibre CSA (*P* < 0.01 and *P* < 0.05 vs. untreated *Gipr* ^+/+^ mice, respectively).

**Conclusions:**

GIP promotes the differentiation of muscle FAPs into adipocytes and its receptor antagonism suppresses IMAT accumulation and promotes muscle regeneration. Pharmacological GIP receptor antagonism may serve as a novel therapeutic approach for sarcopenia.

## Introduction

Sarcopenia is a syndrome of progressive and generalized decline in skeletal muscle mass with age, accompanied by low muscle strength and/or low physical performance, as defined by the European Working Group on Sarcopenia in Older People (EWGSOP) and the Asia Working Group for Sarcopenia (AWGS).^1^, ^2^ With progressive population aging, the prevalence of sarcopenia will further increase in most geriatric settings worldwide. Importantly, sarcopenia is closely related to increased adverse outcomes such as falls, frailty, physical disability and mortality.^3^ Accordingly, exploring the detailed molecular mechanisms underlying sarcopenia pathophysiology and designing novel preventive strategies for this syndrome will be required to promote healthy longevity.

Gastric inhibitory polypeptide (GIP) is a 42‐amino‐acid hormone that is released from intestinal K cells in response to ingestion of nutrients including fat or glucose and potentiates glucose‐dependent insulin secretion from pancreatic β‐cells.^4^ The GIP receptor is expressed in adipocytes and its receptor signalling directly induces fatty acid incorporation into adipose tissue.^5^ Besides, we previously reported that GIP receptor‐deficient mice fed a high‐fat diet did not develop obesity or insulin resistance.^6^, ^7^ However, it is unclear whether GIP receptor signalling promotes intramuscular fat accumulation as an extra‐pancreatic action.

Intramuscular adipose tissue (IMAT) is an ectopic fat accumulation in the interstitium within the muscle (i.e., outside the muscle fibres)^8^, ^9^ and is involved in loss of skeletal muscle strength and physical function.^10^ Its accumulation is known to be enhanced under various conditions such as chronic hyperglycaemia^11^ and aging.^12^, ^13^ IMAT is derived from mesenchymal stem cells, called fibro‐adipogenic progenitors (FAPs), in skeletal muscle,^8^, ^14^ and muscle quality decreases in parallel with the differentiation of FAPs into adipocytes.^15^ Besides intramuscular ectopic fat deposition, FAPs play a crucial role in skeletal muscle regeneration.^15^, ^16^ Indeed, a recent experimental study has reported that FAP‐depleted mice exhibit muscle atrophy and loss of muscle stem cells.^17^ Thus, FAPs appear to exert heterogenous functions according to various pathological conditions.

In the present study, we hypothesized that GIP receptor signalling may be a critical factor determining the differentiation of FAPs into adipocytes and promoting IMAT formation along with declines of muscle mass and strength with aging. To test this hypothesis, we first investigated the alterations of skeletal muscle mass and strength and IMAT formation in aged mice with a genetic deletion of GIP receptor signalling and next explored the molecular mechanisms underlying these alterations, focusing on the differentiation of FAPs into mature adipocytes by GIP and also glycerol‐induced IMAT formation in skeletal muscles of mice with and without a genetic or pharmacological deletion of GIP receptor signalling.

## Materials and methods

### Materials

Reagents and antibodies used in this study are listed with vendors and catalogue numbers in *Table* *S1*.

### Animal studies

GIP receptor (*Gipr*)‐knockout (*Gipr* ^−/−^) mice were generated as previously described,^18^ and male C57BL/6 strain *Gipr* ^−/−^ and wild‐type (*Gipr* ^+/+^) mice were used in this study. For pathophysiological analyses of skeletal muscle and behavioural tests in *Gipr* ^−/−^ mice, the mice were kept with free access to normal diet (CE‐2 [Clea Japan, Tokyo, Japan] or CRF‐1 [Oriental Yeast Co., Ltd., Tokyo, Japan]) and water. For glycerol injection and high‐fat diet studies, the mice were housed with ad libitum access to high‐fat diet (HFD32 comprising 56.7% fat, 23.2% carbohydrate and 20.1% protein; Clea Japan) and water. All animal experiments were approved by the Animal Care and Use Committees of Akita University (protocol numbers a‐1‐0211 and b‐1‐0206, approved 14 May 2020) and Fujita Health University (protocol numbers AP19006 and DP18015, approved 22 February 2019). The experimental animals were handled in accordance with the Animal Welfare Guidelines of Akita University and ARRIVE guidelines.

### Measurements of body composition, plasma triglyceride, grip strength and muscle weight

Whole body composition of 104‐week‐old *Gipr* ^+/+^ and *Gipr* ^−/−^ mice was assessed using micro‐computed tomography (μCT). Mice were anaesthetized with isoflurane and imaged using CosmoScan GX II (Rigaku Corporation, Yamanashi, Japan). A calibrating phantom composed of air and water was scanned along with the animals. Contiguous slice images of the whole body were used for quantitative assessment using Analyze 12.0 (AnalyzeDirect, Overland Park, KS, USA). Weights of bone, lean and adipose tissue masses were determined and normalized by body weight as described previously.^19^ Plasma triglyceride levels were measured on plasma samples collected after a 6‐h daytime fast by an autoanalyser (Fuji Dry‐Chem 5500, Fuji Film, Tokyo, Japan). Grip strength was determined using a model MK‐380Si grip strength metre (Muromachi Kikai, Tokyo, Japan). The values of grip strength were presented as the maximum value repeated 10 times for each mouse as described previously.^20^ To measure muscle weight, the mice were killed using the cervical dislocation method under anaesthesia, and the muscles were dissected immediately. The average weight of bilateral muscles was calculated for the tibialis anterior (TA), extensor digitorum longus, soleus, gastrocnemius and quadriceps femoris muscles in each mouse.

### Immunofluorescence staining and assessment of muscle fibre cross‐sectional area (CSA)

After the mice were killed under anaesthesia, the TA muscles were removed and rapidly frozen. The muscles were cut at a position 2.5 mm from the proximal side, and 10‐μm‐thick muscle tissue sections were prepared for immunofluorescence staining. The specimens were blocked with 1.5% bovine serum albumin for 1 h and incubated with primary antibodies at 4°C overnight followed by secondary staining. Rabbit anti‐perilipin (1:250; Sigma‐Aldrich, St. Louis, MO, USA), rat anti‐laminin (1:400; Santa Cruz Biotechnology, Dallas, TX, USA) and goat anti‐platelet‐derived growth factor receptor alpha (PDGFRα; 1:100; R&D Systems, Minneapolis, MN, USA) antibodies as a primary antibody and Alexa Fluor‐546 anti‐rabbit IgG (1:1000; Invitrogen, Carlsbad, CA, USA), Alexa Fluor‐555 anti‐rat IgG (1:1000; Invitrogen) and Alexa Fluor‐488 anti‐goat IgG (1:1000; Invitrogen) antibodies as a secondary antibody were used. The stained images were obtained using a model BZ‐9000 inverted fluorescence microscope (Keyence, Osaka, Japan). The IMAT area and muscle fibre CSA of the muscle sections were analysed using BZ‐II analyser software Version 2.2 (Keyence). The IMAT area was assessed using perilipin‐stained TA muscle tissues by counting the number of perilipin‐positive cells or by calculating the percentage of perilipin‐positive area for each mouse TA muscle. The muscle fibre CSA was measured using laminin‐stained TA muscle tissues. The strength of the fluorescence signal allowed for the identification of laminin‐positive basal lamina, and an inversion function provided for the identification of muscle fibre. By changing the lower limit in the histogram function, the small, incorrectly identified areas were eliminated. The CSA of each muscle fibre was determined, and then the average CSA was calculated for each mouse.

### In situ hybridization and mRNA expression analysis

For in situ hybridization, the TA muscles were removed from 8‐week‐old *Gipr* ^+/+^ mice. Sense and antisense riboprobes were directed against mouse *Gipr* mRNA (nucleotides 1344–1700; GenBank NM_001080815) and hybridized to the sections as described previously.^21^ For mRNA expression analysis, total RNA was extracted from the TA muscle tissues using the RNeasy Mini Kit (QIAGEN, Hilden, Germany). Complementary DNA (cDNA) was synthesized using a Prime Script First‐Strand cDNA Synthesis Kit (TaKaRa Bio, Shiga, Japan). Quantitative real‐time reverse transcription PCR was performed using a Thermal Cycler Dice Real‐Time System (TaKaRa Bio) and SYBR Premix Ex Taq II (TaKaRa Bio). The relative expression levels of each transcript were calculated as the ratio to 18S ribosomal RNA (rRNA). Primer sequences are listed in *Table* *S2*.

### Cell isolation, purification and culture

Magnetic‐activated cell sorting (MACS) of FAPs was performed as previously described with slight modifications.^22^ Briefly, 12‐week‐old *Gipr* ^+/+^ and *Gipr* ^−/−^ mice were killed under anaesthesia, and the hind limb muscles were carefully dissected to remove the attached tendons and connective tissue. FAPs were isolated from these dissected muscles. To assess the degree of differentiation of the FAPs into mature adipocytes, Oil Red O staining was performed as previously described with slight modifications.^22^ Detailed protocol for cell isolation, purification and culture is described in the supporting information.

### Generation of muscle degeneration models and gastric inhibitory polypeptide receptor antagonist treatment

For the generation of muscle degeneration models, 50 μL of 50% (v/v) glycerol solution in phosphate‐buffered saline (PBS) was injected into bilateral TA muscles of 12‐week‐old *Gipr* ^+/+^ and *Gipr* ^−/−^ mice. The mice were fed a high‐fat diet (HFD32; Clea Japan). On Day 14 after glycerol injection, the mice were killed under anaesthesia. The TA muscles were removed and used for immunofluorescence staining and mRNA expression analysis. For the experiments of GIP receptor antagonist treatment, 12‐week‐old *Gipr* ^+/+^ mice were injected with 50% glycerol bilaterally into the TA muscles as described above. The mice were treated with GIP receptor antagonist, SKL‐14959 (Sanwa Kagaku, Mie, Japan), for 14 days. Untreated *Gipr* ^+/+^ mice served as the control group. The SKL‐14959 was administered at 0.133% in a mixed HFD32 (Clea Japan) as described previously.^23^ Amount of daily food intake was measured during the treatment. After 14‐day administration of SKL‐14959, the mice were killed under anaesthesia, and the removed TA muscles were used for immunofluorescence staining and mRNA and protein expression analyses. The protein expression levels of adipogenesis‐related proteins were analysed by western blot. Detailed protocol for western blot analysis is described in the supporting information.

### Evaluation of behaviour

Behavioural tests were performed at 10–20 weeks of age in *Gipr* ^+/+^ and *Gipr* ^−/−^ mice as described previously.^24^ For the experiments of GIP receptor antagonist treatment, the tests were started after 3‐day administration of SKL‐14959 at 0.133% in a mixed high‐fat diet (HFD32) to *Gipr* ^+/+^ mice at 9–10 weeks of age. Detailed protocol for behavioural tests is described in the supporting information.

### Statistical analysis

All data are presented as mean ± SEM. Statistical analysis was performed using GraphPad Prism 9 software (GraphPad, San Diego, CA, USA). The significance of the difference between the two groups was assessed using two‐tailed Student's *t*‐test. Differences between multiple groups were determined using one‐way analysis of variance (ANOVA) followed by Bonferroni's multiple comparison test. Behavioural data were analysed using two‐way repeated measures ANOVA or two‐tailed Student's *t*‐test. Food intake data were analysed by two‐way repeated measures ANOVA. Statistical significance was set at *P* < 0.05.

## Results

### Aged *Gipr* ^−/−^ mice exhibit a greater proportion of lean tissue mass and increases in muscle strength and mass

**Figure 1:**
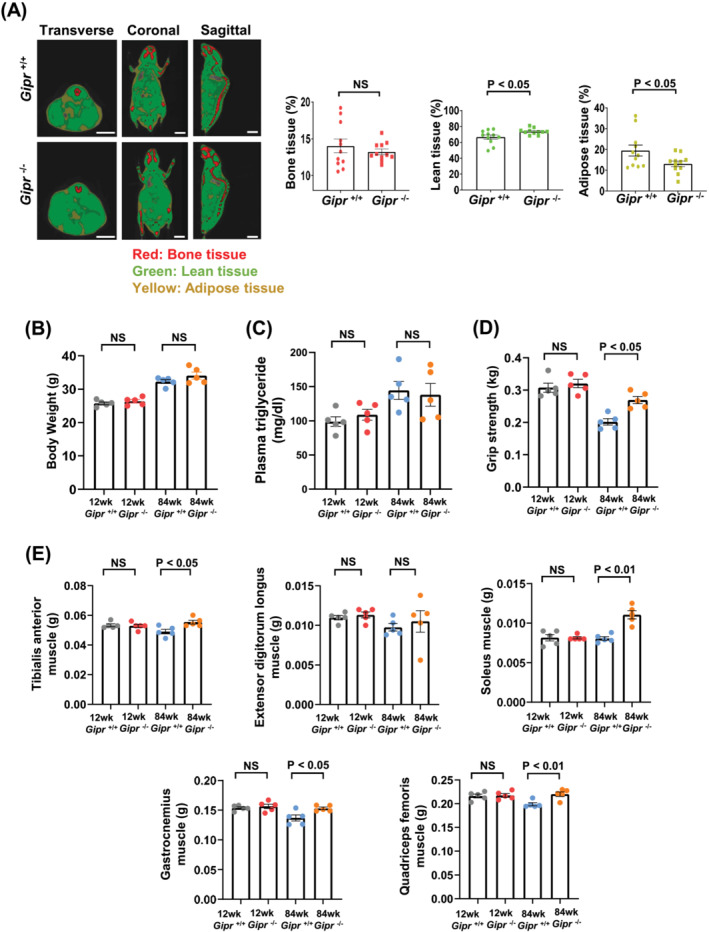
Aged *Gipr* ^−/−^ mice exhibit a greater proportion of lean tissue mass and increases in muscle strength and mass. (A) Representative micro‐computed tomography images of whole body (*left*) and quantitative analysis of body composition (*right*) of 104‐week‐old *Gipr* ^+/+^ and *Gipr* ^−/−^ mice. The red, green and yellow areas represent bone tissue, lean tissue and adipose tissue, respectively. Bars indicate 1 cm. Data were normalized to body weight. *n* = 11 mice per group. (B) Body weight, (C) plasma triglyceride, (D) grip strength and (E) weights of the tibialis anterior, extensor digitorum longus, soleus, gastrocnemius and quadriceps femoris muscles of 12‐week‐old *Gipr* ^+/+^ and *Gipr* ^−/−^ mice and 84‐week‐old *Gipr* ^+/+^ and *Gipr* ^−/−^ mice. The muscle weights are shown as the average value of bilateral muscle weights in each mouse. *n* = 5 mice per group. All data are presented as mean ± SEM. NS, not significant.

Body composition analysis by μCT revealed that 104‐week‐old *Gipr* ^−/−^ mice had a greater proportion of lean tissue mass (non‐bone fat‐free mass) (73.7 ± 1.2% vs. 66.5 ± 2.7%, *P* < 0.05 vs. 104‐week‐old *Gipr* ^+/+^ mice) and less adipose tissue mass (13.1 ± 1.3% vs. 19.4 ± 2.6%, *P* < 0.05 vs. 104‐week‐old *Gipr* ^+/+^ mice) (*Figure* *1* *A*). No statistically significant differences in body weight and plasma triglyceride levels were evident between *Gipr* ^+/+^ and *Gipr* ^−/−^ mice at 12 and 84 weeks of age (*Figure* *1* *B,C*). Although the grip strength of 12‐week‐old *Gipr* ^+/+^ and *Gipr* ^−/−^ mice was not different (0.307 ± 0.014 vs. 0.320 ± 0.013 kg, not significant), an increase in grip strength was observed in 84‐week‐old *Gipr* ^−/−^ mice compared with 84‐week‐old *Gipr* ^+/+^ mice (0.269 ± 0.011 vs. 0.202 ± 0.010 kg, *P* < 0.05) (*Figure* *1* *D*). The masses of the TA, soleus, gastrocnemius and quadriceps femoris muscles, but not extensor digitorum longus muscle, of 84‐week‐old *Gipr* ^−/−^ mice were significantly increased as compared with those of 84‐week‐old *Gipr* ^+/+^ mice (*Figure* *1* *E*).

### Aged *Gipr* ^−/−^ mice show a greater muscle fibre diameter and a lesser intramuscular adipose tissue area in tibialis anterior muscle

**Figure 2:**
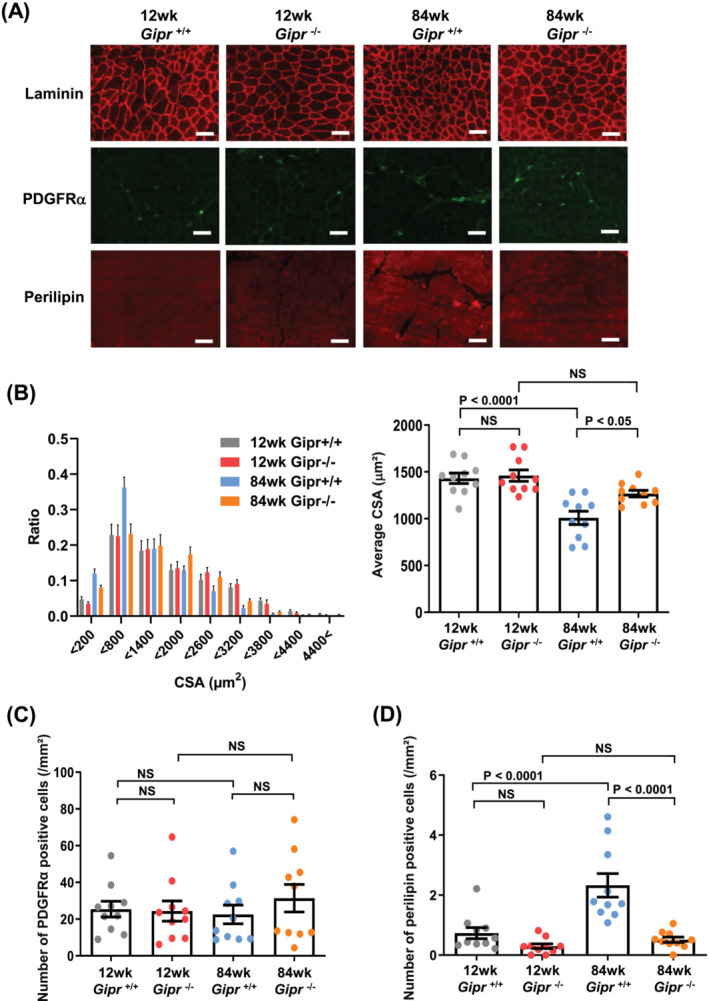
Aged *Gipr* ^−/−^ mice show a greater muscle fibre diameter and a lesser intramuscular adipose tissue area in tibialis anterior (TA) muscle. (A) Representative images of laminin, platelet‐derived growth factor receptor alpha (PDGFRα) and perilipin‐stained TA muscle tissues in 12‐week‐old *Gipr* ^+/+^ and *Gipr* ^−/−^ mice and 84‐week‐old *Gipr* ^+/+^ and *Gipr* ^−/−^ mice. Bars indicate 100 μm. (B) Distribution of muscle fibre cross‐sectional area (CSA) (*left*) and average muscle fibre CSA (*right*) measured using laminin‐stained TA muscle tissues. *n* = 10 TA muscles from 5 mice per group. (C) Number of PDGFRα‐positive cells and (D) number of perilipin‐positive cells per square millimetres of TA muscle tissues. *n* = 10 TA muscles from 5 mice per group. All data are presented as mean ± SEM. NS, not significant.

Quantification of muscle fibre CSA in laminin‐stained TA muscle tissues revealed that average muscle fibre diameter in *Gipr* ^+/+^ mice decreased with aging (*Figure* *2* *A,B*). However, the decline in muscle fibres was attenuated in 84‐week‐old *Gipr* ^−/−^ mice (*Figure* *2* *A,B*). PDGFRα‐positive cells, which are synonymous with FAPs in the TA muscle, were not significantly different among the groups (*Figure* *2A,C*). Perilipin is a major coat protein on the surfaces of lipid droplets in adipocytes, and it is used as a specific marker for lipid accumulation.^25^ We assessed the IMAT area in the TA muscles of *Gipr* ^+/+^ and *Gipr* ^−/−^ mice by counting the number of perilipin‐positive cells. The IMAT areas of 12‐week‐old *Gipr* ^+/+^ and *Gipr* ^−/−^ mice were not significantly different (*Figure* *2A,D*). However, the IMAT area of 84‐week‐old *Gipr* ^+/+^ mice was larger than that of 12‐week‐old *Gipr* ^+/+^ mice (*Figure* *2A,D*). Eighty‐four‐week‐old *Gipr* ^−/−^ mice displayed a reduction in IMAT area compared with 84‐week‐old *Gipr* ^+/+^ mice (*Figure* *2A,D*).

### *Gipr* ^−/−^ mice exhibit slightly increased locomotor activity, decreased anxiety‐like behaviour and improved spatial learning and memory ability

**Figure 3:**
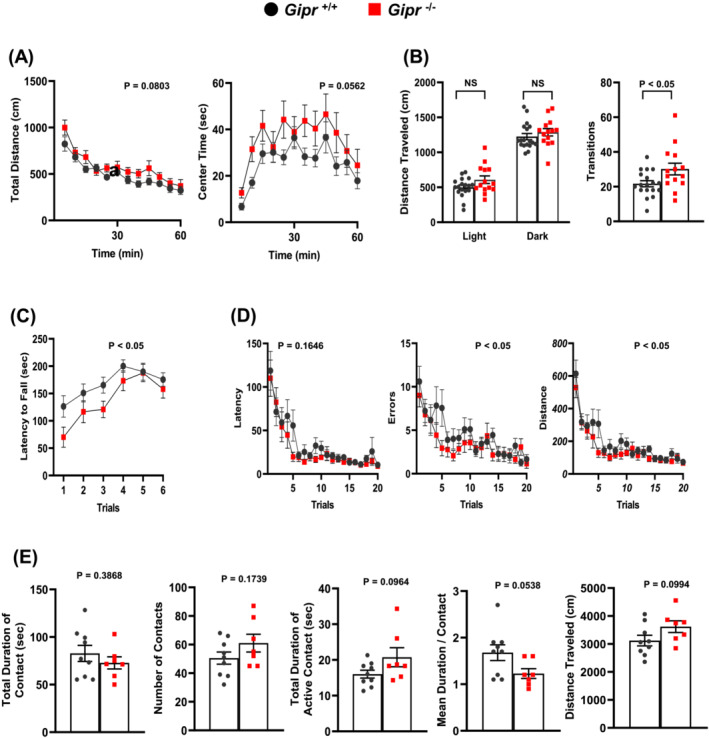
*Gipr* ^−/−^ mice exhibit slightly increased locomotor activity, decreased anxiety‐like behaviour and improved spatial learning and memory ability. The behavioural tests were performed at 10–20 weeks of age in *Gipr* ^+/+^ and *Gipr* ^−/−^ mice. (A) Open field test, (B) light/dark transition test, (C) rotarod test, (D) Barnes maze test and (E) social interaction test. *n* = 18 for *Gipr* ^+/+^ mice and *n* = 14 for *Gipr* ^−/−^ mice (A–D). *n* = 9 for pairs of *Gipr* ^+/+^ mice and *n* = 7 for pairs of *Gipr* ^−/−^ mice (E). All data are presented as mean ± SEM. NS, not significant. *P* values indicate interaction between two factors of *Gipr* ^+/+^ or *Gipr* ^−/−^ and time or trials (A, C, D).

We also examined behavioural phenotype including motor functions and emotional behaviours at 10–20 weeks of age in *Gipr* ^+/+^ and *Gipr* ^−/−^ mice. Total distance and centre time in the open field test tended to be increased in *Gipr* ^−/−^ mice compared with *Gipr* ^+/+^ mice (*Figure* *3* *A*). In the light/dark transition test, *Gipr* ^−/−^ mice exhibited increased number of transitions between the dark and light chambers (*Figure* *3* *B*). *Gipr* ^−/−^ mice initially fell off the rotarod in a shorter time than *Gipr* ^+/+^ mice, but they could stay on the rod as well as *Gipr* ^+/+^ mice after the number of trials was repeated (*Figure* *3* *C*). In the Barnes maze test, the number of errors and distance travelled to reach the target hole was significantly decreased in *Gipr* ^−/−^ mice compared with *Gipr* ^+/+^ mice (*Figure* *3* *D*). In the social interaction test, *Gipr* ^−/−^ mice tended to increase total duration of active contacts and distance travelled and decrease mean duration of contacts compared with *Gipr* ^+/+^ mice (*Figure* *3* *E*). Thus, *Gipr* ^−/−^ mice exhibited slightly increased locomotor activity, decreased anxiety‐like behaviour and improved spatial learning and memory ability relative to *Gipr* ^+/+^ mice.

### Gastric inhibitory polypeptide promotes adipogenic differentiation of fibro‐adipogenic progenitors

**Figure 4:**
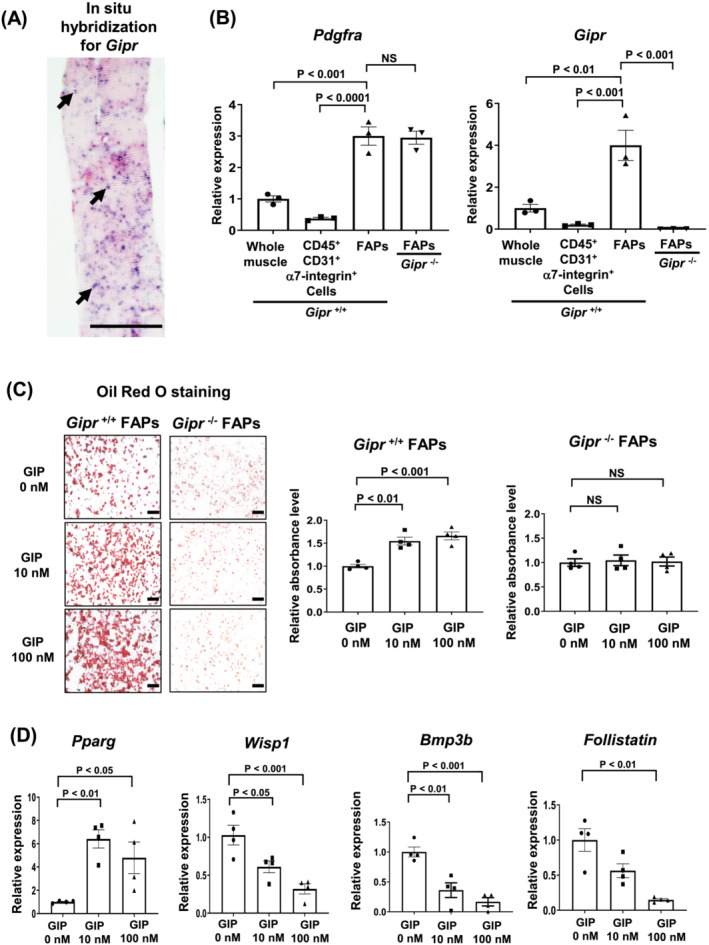
Gastric inhibitory polypeptide (GIP) promotes adipogenic differentiation of fibro‐adipogenic progenitors (FAPs) isolated from lower limb muscles of *Gipr* ^+/+^ mice. (A) In situ hybridization analysis using antisense riboprobes of mouse *Gipr* mRNA in tibialis anterior muscle of 8‐week‐old *Gipr* ^+/+^ mouse. Arrows indicate *Gipr* mRNA expression. Bar indicates 100 μm. (B) Relative mRNA expression levels of *Pdgfra* and *Gipr* in whole muscle, CD45^+^, CD31^+^ or α7‐integrin^+^ cells, and FAPs in lower limb muscle of 12‐week‐old *Gipr* ^+/+^ mouse, and FAPs in lower limb muscle of 12‐week‐old *Gipr* ^−/−^ mouse. *n* = 3 mice per group. (C) Representative images (*left*) and relative absorbance levels at 492 nm (*right*) in Oil Red O staining in FAPs isolated from lower limb muscles of 12‐week‐old *Gipr* ^+/+^ and *Gipr* ^−/−^ mice and cultured under the conditions of absence (0 nM) and presence (10 or 100 nM) of GIP. The absorbance at 492 nm denotes the effectiveness of adipogenic differentiation of FAPs. Bars indicate 100 μm. *n* = 4 mice per group. (D) Relative mRNA expression levels of *Pparg*, *Wisp1*, *Bmp3b* and *follistatin* in FAPs isolated from lower limb muscles of 12‐week‐old *Gipr* ^+/+^ mice and cultured under the conditions of absence (0 nM) and presence (10 or 100 nM) of GIP. *n* = 4 mice per group. All data are presented as mean ± SEM. NS, not significant.

In situ hybridization analysis in 8‐week‐old *Gipr* ^+/+^ mice indicated that *Gipr* mRNA was morphologically expressed around muscle fibres, that is, in the interstitium, within the TA muscle (*Figure* *4* *A*). To assess whether the detected *Gipr* gene expression originated from FAPs, we isolated FAPs from lower limb muscles of 12‐week‐old *Gipr* ^+/+^ and *Gipr* ^−/−^ mice using MACS as CD45^−^, CD31^−^, α7‐integrin^−^ and Sca‐1^+^ cells. Indeed, the isolated FAPs were highly enriched for the expression of *Pdgfra*, which is generally used as a cell marker of FAPs,^26^ and the expression levels were not significantly different between FAPs from *Gipr* ^+/+^ and *Gipr* ^−/−^ mice (*Figure* *4* *B*). The FAPs isolated from *Gipr* ^+/+^ mice exhibited high gene expression levels of *Gipr* (*Figure* *4* *B*). In contrast, *Gipr* expression levels in the CD45^+^, CD31^+^ and α7‐integrin^+^ fraction consisted of immune, endothelial and satellite cells, respectively, were considerably lower than those in the fraction of FAPs in *Gipr* ^+/+^ mice (*Figure* *4* *B*).

To investigate whether GIP promoted the differentiation of FAPs into mature adipocytes, we performed Oil Red O staining in the FAPs isolated from lower limb muscles of *Gipr* ^+/+^ and *Gipr* ^−/−^ mice and cultured under the conditions of absence (0 nM) and presence (10 or 100 nM) of GIP, followed by the semi‐quantitative analysis by measurement of absorbance at 492 nm, which denotes the effectiveness of adipogenic differentiation of FAPs (*Figure* *4* *C*). The addition of 10 and 100 nM of GIP in *Gipr* ^+/+^ FAPs promoted adipogenesis 1.6‐ and 1.7‐fold as compared with the lack of GIP (*P* < 0.01 and *P* < 0.001 vs. 0 nM of GIP, respectively), whereas the GIP addition in *Gipr* ^−/−^ FAPs did not facilitate adipogenesis (*Figure* *4* *C*).

Besides, the mRNA (*Pparg*) expression levels of peroxisome proliferator‐activated receptor gamma (PPARγ), which is a prominent regulator of adipogenesis,^27^ were significantly increased by the GIP addition (*Figure* *4* *D*). Undifferentiated FAPs have been recently reported to secrete favourable factors for efficient myogenesis, such as WNT1‐inducible signalling pathway protein 1 (WISP1), bone morphogenetic protein 3B (BMP3B) and follistatin.^28^, ^29^, ^30^ The mRNA (*Wisp1*, *Bmp3b* and *follistatin*) expression levels of WISP1, BMP3B and follistatin in FAPs were significantly reduced along with adipogenic differentiation following GIP addition (*Figure* *4* *D*).

### Gastric inhibitory polypeptide receptor signalling is involved in adipogenesis following glycerol injury

**Figure 5:**
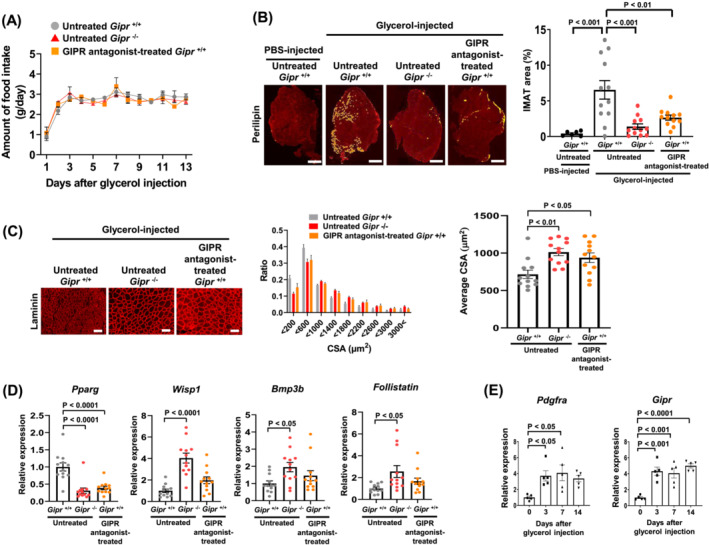
Gastric inhibitory polypeptide receptor (GIPR) signalling is involved in adipogenesis following glycerol injury. (A) Changes in amount of daily food intake in glycerol‐injected and untreated *Gipr* ^+/+^ and *Gipr* ^−/−^ mice and glycerol‐injected and GIPR antagonist‐treated *Gipr* ^+/+^ mice. Twelve‐week‐old mice were either treated or untreated with GIPR antagonist for 14 days after glycerol injection into the tibialis anterior (TA) muscles. All mice were fed with a high‐fat diet for 14 days. *n* = 6 mice per group. (B) Representative images (*left*) and intramuscular adipose tissue (IMAT) areas (*right*) of the TA muscle tissue sections stained with perilipin antibodies on Day 14 after phosphate‐buffered saline (PBS) or glycerol injection into the TA muscles of 12‐week‐old *Gipr* ^+/+^ and *Gipr* ^−/−^ mice. Bars indicate 500 μm. *n* = 6 TA muscles from 3 mice in PBS‐injected mouse group. *n* = 12 TA muscles from 6 mice per glycerol‐injected mouse group. (C) Representative images (*left*), distribution of muscle fibre cross‐sectional area (CSA) (*centre*) and average muscle fibre CSA (*right*) analysed using laminin‐stained TA muscle tissues on Day 14 after glycerol injection into the TA muscles of 12‐week‐old *Gipr* ^+/+^ and *Gipr* ^−/−^ mice. Bars indicate 100 μm. *n* = 12 TA muscles from 6 mice per group. (D) Relative *Pparg*, *Wisp1*, *Bmp3b* and *follistatin* expression levels of mRNA extracted from the TA muscles on Day 14 after glycerol injection into the TA muscles of 12‐week‐old *Gipr* ^+/+^ and *Gipr* ^−/−^ mice. *n* = 12 TA muscles from 6 mice per group. (E) Relative mRNA expression levels of *Pdgfra* and *Gipr* in the TA muscles on Days 0, 3, 7 and 14 after glycerol injection into the TA muscles of 12‐week‐old *Gipr* ^+/+^ mice. *n* = 5 TA muscles from 5 mice per group. All data are presented as mean ± SEM.

Because an intramuscular glycerol injection induces IMAT development and disturbs muscle regeneration^31^ and a high‐fat diet promotes the accumulation of subcutaneous and visceral fat,^6^ their combination is expected to promote IMAT accumulation to a greater extent. Therefore, we performed glycerol injection into the TA muscles of 12‐week‐old *Gipr* ^+/+^ and *Gipr* ^−/−^ mice followed by a high‐fat diet for 14 days or administration of GIP receptor antagonist at 0.133% in a mixed high‐fat diet for 14 days and examined IMAT accumulation and muscle regeneration. Amount of daily food intake was not different among the untreated *Gipr* ^+/+^, untreated *Gipr* ^−/−^ and GIP receptor antagonist‐treated *Gipr* ^+/+^ mouse groups (*Figure* *5* *A*). *Figure* *5* *B* shows representative perilipin staining images and IMAT area of the TA muscles on Day 14 after glycerol injection or PBS injection as a control. Glycerol‐injected and untreated *Gipr* ^+/+^ mice exhibited greater infiltration of adipocytes expressing perilipin on the surface of lipid droplets relative to PBS‐injected and untreated *Gipr* ^+/+^ mice (6.54 ± 1.30 % vs. 0.42 ± 0.13 %, *P* < 0.001) (*Figure* *5* *B*). In comparison, glycerol‐injected and untreated *Gipr* ^−/−^ mice showed less adipogenesis (IMAT area) than glycerol‐injected and untreated *Gipr* ^+/+^ mice (1.39 ± 0.38 % vs. 6.54 ± 1.30 %, *P* < 0.001) (*Figure* *5* *B*). Besides, glycerol‐injected and GIP receptor antagonist‐treated *Gipr* ^+/+^ mice exhibited less IMAT area relative to glycerol‐injected and untreated *Gipr* ^+/+^ mice (2.65 ± 0.36 % vs. 6.54 ± 1.30 %, *P* < 0.01) (*Figure* *5* *B*). Quantification analysis in laminin‐stained muscle tissues revealed that the average CSA of TA muscle fibres in untreated *Gipr* ^−/−^ mice was nearly 1.5‐fold larger than that in untreated *Gipr* ^+/+^ mice (*P* < 0.01) and also that the average TA muscle fibre CSA in GIP receptor antagonist‐treated *Gipr* ^+/+^ mice was significantly increased as compared with that in untreated *Gipr* ^+/+^ mice (*P* < 0.05) (*Figure* *5* *C*). The mRNA expression levels of an adipogenesis regulator *Pparg* in the TA muscles were significantly lower in untreated *Gipr* ^−/−^ mice and GIP receptor antagonist‐treated *Gipr* ^+/+^ mice than in untreated *Gipr* ^+/+^ mice after glycerol injection (*Figure* *5* *D*). In contrast, the mRNA expression levels of myogenesis promotors such as *Wisp1*, *Bmp3b* and *follistatin* in the TA muscles were significantly higher in untreated *Gipr* ^−/−^ mice than in untreated *Gipr* ^+/+^ mice (*Figure* *5* *D*). The mRNA expression levels of *Wisp1*, *Bmp3b* and *follistatin* in the TA muscles tended to be increased in GIP receptor antagonist‐treated *Gipr* ^+/+^ mice relative to untreated *Gipr* ^+/+^ mice, although their differences did not reach statistical significance (*Figure* *5* *D*).

Next, we tested the gene expression in the TA muscles prior to and 3, 7 and 14 days following glycerol injection in 12‐week‐old *Gipr* ^+/+^ mice. The mRNA expression levels of *Pdgfra*, a cell marker of FAPs, were increased following glycerol‐induced muscle injury as compared with those before injury (*Figure* *5* *E*). In addition, *Gipr* mRNA expression levels were increased following glycerol‐induced muscle injury, as were *Pdgfra* expression levels (*Figure* *5* *E*). Thus, we confirmed that glycerol injection enhanced gene expression of GIP receptor along with a cell marker of FAPs, PDGFRα, in the TA muscles, both of which could be involved in IMAT formation.

### Gastric inhibitory polypeptide receptor antagonist decreases adipogenesis‐related protein expression levels in tibialis anterior muscle following glycerol injury

**Figure 6:**
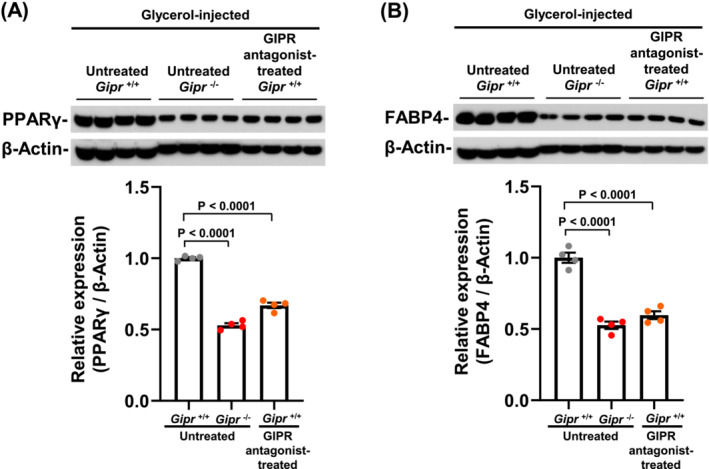
Gastric inhibitory polypeptide receptor (GIPR) antagonist decreases adipogenesis‐related protein expression levels in tibialis anterior (TA) muscle following glycerol injury. Western blot analyses for peroxisome proliferator‐activated receptor gamma (PPARγ) (A) and fatty acid binding protein 4 (FABP4) (B) in the TA muscles on Day 14 after glycerol injection into the TA muscles of 12‐week‐old *Gipr* ^+/+^ and *Gipr* ^−/−^ mice. Upper and lower panels show representative western blots and their quantitative data, respectively. *n* = 4 TA muscles from 4 mice per group. All data are presented as mean ± SEM.

As PPARγ and fatty acid binding protein 4 (FABP4) are involved in IMAT formation as an adipogenesis‐related protein, we investigated their protein expression levels in the TA muscles of untreated *Gipr* ^+/+^ and *Gipr* ^−/−^ mice and GIP receptor antagonist‐treated *Gipr* ^+/+^ mice for 14 days after glycerol injection into the TA muscles. The PPARγ and FABP4 protein expression levels were significantly lower in the untreated *Gipr* ^−/−^ and GIP receptor antagonist‐treated *Gipr* ^+/+^ mouse groups than in the untreated *Gipr* ^+/+^ mouse group (*Figure* *6* *A,B*).

### Gastric inhibitory polypeptide receptor antagonist increases locomotor activity

**Figure 7:**
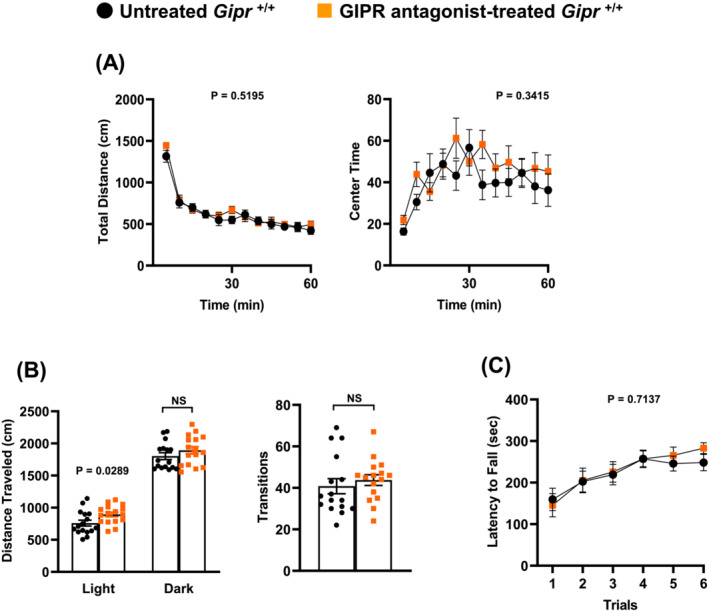
Gastric inhibitory polypeptide receptor (GIPR) antagonist increases locomotor activity. (A) Open field test, (B) light/dark transition test and (C) rotarod test. The behavioural tests were started after 3‐day administration of GIPR antagonist in a mixed high‐fat diet to *Gipr* ^+/+^ mice at 9–10 weeks of age. *n* = 16 for untreated control *Gipr* ^+/+^ mice and *n* = 16 for GIPR antagonist‐treated *Gipr* ^+/+^ mice. All data are presented as mean ± SEM. NS, not significant. *P* values indicate interaction between two factors of untreated *Gipr* ^+/+^ or GIPR antagonist‐treated *Gipr* ^+/+^ and time or trials (A, C). NS, not significant.

Behavioural tests were started after 3‐day administration of GIP receptor antagonist in a mixed high‐fat diet to *Gipr* ^+/+^ mice at 9–10 weeks of age. There was no difference in behavioural phenotype evaluated by open field test and rotarod test between the untreated control and GIP receptor antagonist‐treated groups (*Figure* *7* *A,C*). In light/dark transition test, the GIP receptor antagonist‐treated group exhibited a significant increase in distance travelled in light chamber, indicating that GIP receptor antagonist increases locomotor activity (*Figure* *7* *B*).

## Discussion

The present study was conducted to explore the role of GIP receptor signalling in age‐related declines of skeletal muscle mass and strength and the molecular mechanisms underlying these alterations and to evaluate the therapeutic potential of its blockade for sarcopenia treatment. We previously reported that 50‐week‐old *Gipr* ^−/−^ C57BL/6 mice exhibited a greater proportion of lean tissue mass than *Gipr* ^+/+^ mice.^7^ Zhu et al. reported decreased muscle mass and increased IMAT as a phenotype of sarcopenia in 72‐ to 88‐week‐old *Gipr* ^+/+^ C57BL/6 mice.^32^ However, it is unclear whether older *Gipr* ^−/−^ mice exhibit greater muscle mass and strength than older *Gipr* ^+/+^ mice. Notably, we here demonstrated that 84‐week‐old *Gipr* ^+/+^ mice showed a loss of muscle mass and weakened muscle strength with aging, whereas 84‐week‐old *Gipr* ^−/−^ mice did not display muscle mass loss with aging. Moreover, μCT analysis revealed that the lean tissue mass of the whole body in 104‐week‐old *Gipr* ^−/−^ mice was greater than that in 104‐week‐old *Gipr* ^+/+^ mice. These findings suggest that GIP receptor signalling is closely associated with age‐related changes in skeletal muscle.

It is appreciated that IMAT is a pathological feature of muscle in sarcopenia.^33^ This agrees with our observation of the increased IMAT area, as evidenced by the large number of perilipin‐positive cells, in 84‐week‐old *Gipr* ^+/+^ mice showing decreased average muscle fibre diameter as compared with 12‐week‐old *Gipr* ^+/+^ mice. Besides, average muscle fibre diameter was increased in 84‐week‐old *Gipr* ^*−/−*^ mice compared with 84‐week‐old *Gipr* ^*+/+*^ mice. Given the evidence showing that the increase in muscle fibre diameter reflects the degree of muscle fibre regeneration,^34^ our data indicate that muscle fibre regeneration in *Gipr* ^*−/−*^ mice is more effective than that in *Gipr* ^*+/+*^ mice, suggesting the beneficial action of GIP receptor signalling blockade in muscle fibre regeneration. Regarding increases in muscle mass and strength via such enhanced muscle fibre regeneration, elevated physical activity also might affect them. Indeed, it has been reported that 24‐week‐old *Gipr* ^−/−^ mice show increased physical activity compared with 24‐week‐old *Gipr* ^+/+^ mice.^35^ Similarly, the present results demonstrated that *Gipr* ^−/−^ mice display higher locomotor activity caused by lower anxiety‐like behaviour and/or stronger desire for exploration and improved spatial learning and memory ability than *Gipr* ^+/+^ mice at 10–20 weeks of age. This may be attributed to either the direct action of GIP receptor signalling blockade on the brain or the enhanced glucagon‐like peptide‐1 action in the brain by blocking the GIP action, as occurs in pancreatic β‐cells.^36^ Unfortunately, the present study did not reveal whether such elevated physical activity affected increases in muscle mass and strength or whether increased muscle mass and strength contributed to physical activity enhancement in the aged *Gipr* ^*−/−*^ mice. To address this issue, further studies would be required.

Importantly, the concentration of plasma total GIP in *Gipr* ^+/+^ mice has been reported to gradually increase with aging.^37^ Furthermore, such increased GIP seems to be closely linked to adipogenesis with aging. Several direct adipocyte actions of GIP such as stimulation of adipogenesis and enhancement of lipoprotein lipase (LPL) have been identified. GIP enhances the activity of LPL on the cell surface of adipocytes that hydrolyses lipoprotein‐associated triglycerides to produce free fatty acids available for local uptake.^38^ Free fatty acids and their derivatives act as ligands of PPARγ, which ultimately leads to the differentiation into mature adipocytes.^27^ Given these lines of evidence, we hypothesized that FAPs of *Gipr* ^+/+^ mice may be promoted to differentiate into mature adipocytes by GIP receptor signalling, whereas FAPs of *Gipr* ^−/−^ mice may maintain undifferentiated mesenchymal precursors. Expectedly, we observed that GIP addition to FAPs isolated from lower limb muscles of *Gipr* ^+/+^ mice in vitro contributed to an increase in lipid droplets and an elevation in *Pparg* mRNA expression levels in adipogenically differentiated FAPs. This novel finding provides the first evidence that GIP receptor signalling operates similarly in adipocytes from common adipose tissue and FAPs. Factors synthesized by FAPs such as WISP1, BMP3B and follistatin support the differentiation of myogenic progenitors into mature muscle fibres.^28^, ^29^, ^30^ It is noteworthy that *Wisp1*, *Bmp3b* and *follistatin* mRNA expression of FAPs was significantly downregulated along with GIP‐mediated promotion of FAPs to the differentiation into mature adipocytes in our in vitro study. Besides, we confirmed that muscle *Wisp1*, *Bmp3b* and *follistatin* mRNA expression levels were higher in *Gipr* ^−/−^ mice than in *Gipr* ^+/+^ mice following glycerol injection into the TA muscles. Of the upregulated myogenic factors, follistatin has been well studied for its role in the regulation of muscle growth as an antagonist of myostatin that inhibits muscle growth. Indeed, transgenic mice overexpressing follistatin display increased muscle mass.^39^ Furthermore, in a clinical trial involving patients with Becker muscular dystrophy treated with follistatin delivered by an adeno‐associated virus, increased muscle fibre size distribution with muscle hypertrophy was noted, and no adverse effects were apparent.^40^ Considering that IMAT originates from the differentiation of FAPs into mature adipocytes,^26^ the present findings collectively indicate that GIP contributes to the formation of IMAT and impairs muscle fibre regeneration by downregulating myogenesis factors such as WISP1, BMP3B and follistatin.

Another novel finding in the present study is that pharmacological blockade of GIP receptor signalling led to reduced IMAT area following glycerol injection into the TA muscles in *Gipr* ^+/+^ mice. Nakamura et al. reported that the GIP receptor antagonist SKL‐14959 decreased triacylglycerol content in the liver and muscles, resulting in suppressed weight gain in diet‐induced obese mice.^23^ We observed that the GIP receptor antagonists reduced the IMAT area and muscle *Pparg* mRNA levels and increased the muscle fibre diameter following glycerol injection into the TA muscles in *Gipr* ^+/+^ mice. Unexpectedly, the GIP receptor antagonists did not significantly upregulate the muscle *Wisp1*, *Bmp3b* and *follistatin* mRNA expression levels. These findings may be attributed to a lower dosage of GIP receptor antagonist administered to the mice. In the present study, like *Gipr* ^−/−^ mice, GIP receptor antagonist treatment increased locomotor activity in *Gipr* ^+/+^ mice, although the treatment term was short. This may indicate that the GIP receptor antagonist contributes to enhancing locomotor activity through promoting muscle fibre regeneration in mice. Taken together, our experimental data suggest that the GIP receptor antagonist treatment may be a novel therapeutic strategy for sarcopenia.

There are several limitations to this study. First, this study investigated the influences of systemic, but not muscle‐specific, GIP receptor deficiency and antagonism on muscle phenotypes. Second, we have not examined the direct effects of favourable factors for efficient myogenesis such as WISP1, BMP3B and follistatin on muscle regeneration. Therefore, further studies are required to support the present findings.

**Figure 8:**
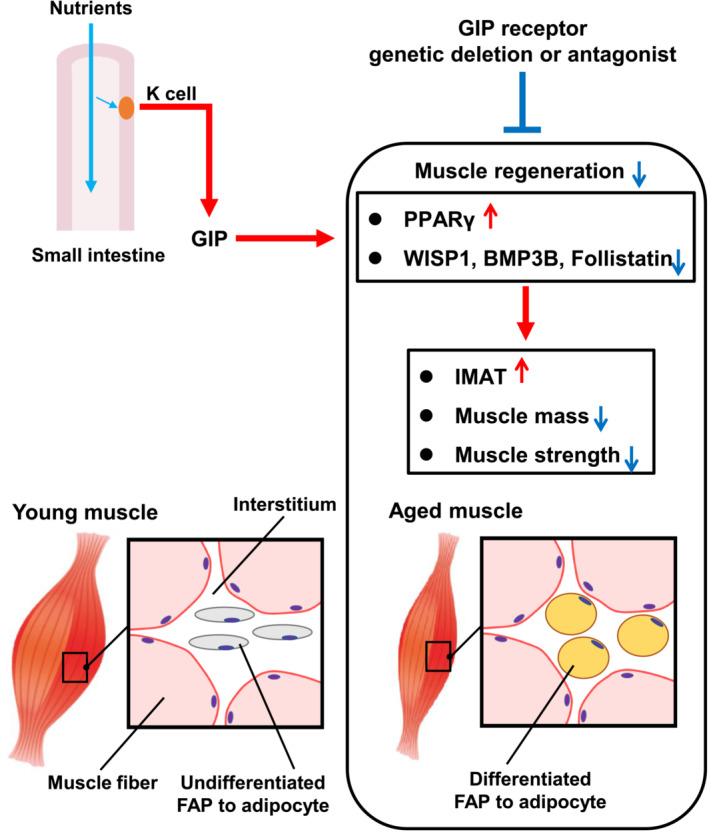
Proposed mechanism of age‐related muscle mass and strength reduction by gastric inhibitory polypeptide (GIP) and muscle protection by inhibition of GIP receptor signalling. BMP3B, bone morphogenetic protein 3B; FAP, fibro‐adipogenic progenitor; IMAT, intramuscular adipose tissue; PPARγ, peroxisome proliferator‐activated receptor gamma; WISP1, WNT1‐inducible signalling pathway protein 1.

In summary, our study demonstrates that GIP receptor signalling could be responsible for the development of sarcopenia accompanied by aging via promoting the differentiation of FAPs into mature adipocytes in skeletal muscles and the following IMAT formation and thereby reducing muscle regeneration (*Figure* *8*). Furthermore, it is likely that blockade of GIP receptor signalling facilitates an increase in muscle mass without any adverse influence on cognitive function and physical activity. Finally, the present findings suggest that pharmacological GIP receptor antagonism may serve as a novel therapeutic approach for sarcopenia.

## Conflict of interest statement

The authors declare no conflicts of interest.

## Supporting information


**Data S1.** Supporting Information.

**Table S1.** Reagent and antibody list.

**Table S2.** Primer list.
