# Supplementary material for: Gastric inhibitory polypeptide receptor antagonism suppresses intramuscular adipose tissue accumulation and ameliorates sarcopenia
Source: J Cachexia Sarcopenia Muscle. 2023 Oct 27;14(6):2703–18. doi: 10.1002/jcsm.13346 (PMC10751449; doi:10.1002/jcsm.13346)
Supplement: Supplementary file 1 — Data S1. Supporting Information. [file JCSM-14-2703-s002.docx]

**Supplementary Methods**

*Cell isolation, purification, and culture*

Muscles were minced with a razor blade and digested with [Collagenase](https://www.sciencedirect.com/topics/biochemistry-genetics-and-molecular-biology/collagenase) Type 2 (Worthington Biochemical Corporation, Lakewood, NJ, USA) in Dulbecco's Modified Eagle Medium (DMEM) with gentle agitation for 45 min at 37°C. Cells were liberated from the digested tissue by five passages using a syringe attached to an 18-gauge needle. The cells were incubated for 15 min at 37°C. The resulting cellular suspension was filtered through a 70 μm cell strainer, pelleted at 300 × g, and washed once with PBS. The Miltenyi MACS purification system (Miltenyi Biotec, Bergisch Gladbach, Germany) was used to isolate FAPs from the muscles. Briefly, cells isolated from digested muscles were incubated with microbead-conjugated anti-CD45 (Miltenyi Biotec), anti-CD31 (Miltenyi Biotec), and anti-α7 integrin (Miltenyi Biotec) for 15 min. Subsequently, the cells were washed once with MACS buffer and pelleted. For cell sorting, the pellet was resuspended in MACS buffer. The cells were passed through a magnetic LD column (Miltenyi Biotec), and the flow-through fraction was CD45^-^/CD31^-^/α7-integrin^-^. This fraction was then incubated with microbead-conjugated anti- stem cell antigen-1 (Sca-1; Miltenyi Biotec) for 15 min. Similarly, we prepared a cell suspension and passed it through a magnetic LS column (Miltenyi Biotec). Finally, the column was removed from the separator, and an appropriate amount of buffer was pipetted onto the column and flushed with magnetically labeled cells. This fraction (CD45^-^/CD31^-^/α7-integrin^-^/Sca-1^+^) was collected for FAPs. The FAPs were cultured in non-coated plastic plates in DMEM supplemented with 10% fetal bovine serum (FBS), basic fibroblast growth factor (bFGF, 2.5 ng/ml), and 1% penicillin/streptomycin, in a humidified tissue culture incubator under standard growth conditions at 37°C and 5% CO_2_. The medium was changed every 48 h to maintain expansion. To induce adipogenic differentiation, the FAPs were incubated in DMEM containing 10% FBS, insulin (5 μg/ml), dexamethasone (1 μM), and isobutylmethylxanthine (50 mM) for 2 days. After then, the FAPs were cultured in DMEM containing either vehicle (PBS), 10 nM GIP (1-42; AnaSpec, Fremont, CA, USA), or 100 nM GIP (1-42; AnaSpec) in addition to 10% FBS and insulin (5 μg/ml) for 6 days, and the cell samples were subjected to Oil Red O staining and mRNA expression analysis. The mRNA expression analysis in the cell samples was performed according to the same protocol as that in the TA muscle tissues. To assess the degree of differentiation of the FAPs into mature adipocytes, Oil Red O staining was performed. Briefly, the cells were fixed in 10% formalin for 30 min, stained with Oil Red O in 60% isopropanol for 10 min, and counterstained with DAPI. The adipogenic efficiency was calculated by dividing the relative absorbance at 492 nm by the number of cells.

*Western blot analysis*

Tibialis anterior (TA) muscle tissues were homogenized in lysis buffer (0.25 M sucrose, 10 mM Tris, 1 mM EDTA, pH 7.4), centrifuged at 10,000 × g for 10 min at 4°C, and the cleared lysate was used for Western blot analysis. The muscle proteins were separated by SDS-PAGE using NuPAGETM 10% Bis-Tris Gel (Invitrogen, Waltham, MA, USA) and transferred onto polyvinylidene difluoride (PVDF) membrane using iBlot Gel Transfer Stacks (Invitrogen). The PVDF membranes were reacted with rabbit anti-PPARγ polyclonal antibody (1:2,000; Proteintech, Rosemont, IL, USA) and rabbit anti- FABP4 polyclonal antibody (1:5,000; Proteintech), followed by reaction with HRP-conjugated goat anti-rabbit IgG antibody (1:10,000; DakoCytomation, Glostrup, Denmark). The reactions were visualized using an enhanced chemiluminescence detection system (ECL Prime, Amersham, Buckinghamshire, UK). Loading of lysate protein was evaluated by immunoblot using rabbit anti-β-actin antibody (1:1,000; Proteintech) and HRP-conjugated goat anti-rabbit IgG antibody (1:10,000; DakoCytomation). The signal intensity of the protein bands was semiquantified using Adobe Photoshop (version CS5; Adobe Systems, San Jose, CA, USA), and the relative protein expression levels of PPARγ and FABP4 in each muscle tissue sample were calculated as the ratio to β-actin.

*Evaluation of behavior*

Mice were housed in four per cage with ad libitum access to food and water. Behavioral tests were conducted in the following order, starting with the less stressful test; light/dark transition test, open field test, social interaction test, rotarod test, Barnes Maze test. The interval between tests was more than one day. The apparatus of light/dark transition test consisted of a plastic cage (21 × 41.5 × 25 cm; O’Hara & Co, LTD., Tokyo, Japan) that was divide into two chambers of equal size by a partition with a door. One chamber was brightly illuminated (approximately 390 lux), and the other was dark (less than 5 lux). The behavior in the apparatus was recorded over 10 min, and total distance travelled, number of transitions between chambers were analyzed automatically using the ImageLD. The open field test was performed using an open field apparatus (40 × 40 × 30 cm; AccuScan Instruments, Columbus, OH, USA). Each mouse was placed in an open field apparatus, and the total distance traveled, vertical activity, and time spent in the central area were analyzed for 60 min. In the social interaction test, two mice were placed at opposite corners of the open field apparatus (40 × 40 × 30 cm; O’Hara & Co.) and allowed to explore freely for 10 min. The behavior was recorded and analyzed automatically using the ImageSI program. The total duration of contact, number of contacts, total duration of active contact, mean duration per contact, and total distance traveled were measured. An accelerating rotarod (UGO Basile; Comerio, Italy) was used for the rotarod test. Each mouse was placed on rotating drums (3 cm diameter), which were accelerated from 4 to 40 rpm over a 5 min period. The time that each mouse was able to maintain balance on the rotarod was recorded. The Barnes maze task was conducted on dry land using a white circular platform (diameter 1 m) with 12 holes equally spaced around the perimeter (O’Hara & Co.). Twenty trials were performed for each mouse. The number of errors, latency to reach the target, and distance traveled to reach the target were automatically calculated using ImageBM. The ImageLD, ImageSI, and ImageBM used for the behavioral tests were based on Image J program (http://rsb.info.nih.gov/ij /), which were modified for each test by T. Miyakawa.
