# Supplementary material for: Gastric inhibitory polypeptide receptor antagonism suppresses intramuscular adipose tissue accumulation and ameliorates sarcopenia
Source: J Cachexia Sarcopenia Muscle. 2023 Oct 27;14(6):2703–18. doi: 10.1002/jcsm.13346 (PMC10751449; doi:10.1002/jcsm.13346)
Supplement: Supplementary file 2 — Table S1. Reagent and antibody list. [file JCSM-14-2703-s001.docx]

**Supplementary Table 1. Reagent and antibody list**

| **Reagents and Antibodies** | | **SOURCE** | | **IDENRIFIER** | |
| --- | --- | --- | --- | --- | --- |
| Glycerol | | FUJIFILM Wako Pure Chemical Corporation | | Cat#070-04941 | |
| Phosphate Buffered Saline | | Life Technologies Corporation | | Cat#20012-027 | |
| Collagenase Type 2 | | Worthington Biochemical Corporation | | Cat#LS004176 | |
| Dulbecco's Modified Eagle Medium | | Sigma-Aldrich | | Cat#5796 | |
| CD45 MicroBeads, mouse | | Miltenyi Biotec | | Cat#130-052-301 | |
| CD31 MicroBeads, mouse | | Miltenyi Biotec | | Cat#130-097-418 | |
| Anti-Integrin alpha7 MicroBeads, mouse | | Miltenyi Biotec | | Cat#130-104-261 | |
| Anti-Sca-1 MicroBeads (non-HSC), mouse | | Miltenyi Biotec | | Cat#130-106-641 | |
| MACS BSA Stock Solution | | Miltenyi Biotec | | Cat#130-091-376 | |
| autoMACS Rinsing Solution | | Miltenyi Biotec | | Cat#130-091-222 | |
| LD column | | Miltenyi Biotec | | Cat#130-042-901 | |
| LS column | | Miltenyi Biotec | | Cat#130-122-729 | |
| Falcon Cell Strainer 70mm Nylon | | Corning | | Cat#352350 | |
| Fetal Bovine Serum | | Thermo Fisher Scientific | | Cat#12483020 | |
| Mouse FGF-basic Recombinant Protein | | Thermo Fisher Scientific | | Cat#PMG0031 | |
| Penicillin/Streptomycin | | Life Technologies Corporation | | Cat#15140-122 | |
| Insulin | | Sigma-Aldrich | | Cat#12643 | |
| Dexamethasone | | Wako | | Cat#041-18861 | |
| Isobutylmethylxanthine | | Sigma-Aldrich | | Cat#17018 | |
| GIP | | AnaSpec | | Cat#AS-61226-1 | |
| RNeasy Mini Kit | | Qiagen | | Cat#74104 | |
| QIAGEN Proteinase K | | Qiagen | | Cat#19131 | |
| Prime Script First-Strand cDNA Synthesis Kit | | Takara Bio | | Cat#6110A | |
| SYBR Premix Ex tag II | | Takara Bio | | Cat#RR820A | |
| Bovine Serum Albumin | | Sigma-Aldrich | | Cat#A4503 | |
| Perilipin-1 (D1D8) XP® Rabbit mAb | | Sigma-Aldrich | | Cat#9349 | |
| NuPAGE^TM^ 10% Bis-Tris Gel | | invitrogen | | Cat#NP0303Box | |
| iBlot Gel Transfer Stacks | | invitrogen | | Cat#IB401002 | |
| ECL Prime Western Blotting Detection Reagent | | Amersham | | Cat#RPN2232 | |
| PPAR Gamma Polyclonal antibody | | Proteintech | | Cat#16643-1-AP | |
| FABP4 Polyclonal antibody | | Proteintech | | Cat#12802-1-AP | |
| Beta Actin Polyclonal antibody | | Proteintech | | Cat#20536-1-AP | |
| Laminin a-2 (4H8-2) | | Santa Cruz | | Cat#sc-59854 | |
| anti-mPDGF Rα | | R and D Systems | | Cat#AF1062 | |
| Alexa Fluor 546 donkey anti-rabbit IgG (H+L) | | Invitrogen | | Cat#A10040 | |
| Alexa Fluor 555 goat anti-rat IgG (H+L) | | Invitrogen | | Cat#A21434 | |
| Alexa Fluor 488 donkey anti-goat IgG (H+L) | | Invitrogen | | Cat#A11078 | |
| Oil red O | | Sigma-Aldrich | | Cat#O0625 | |
