# Supplementary material for: Gastric inhibitory polypeptide receptor antagonism suppresses intramuscular adipose tissue accumulation and ameliorates sarcopenia
Source: J Cachexia Sarcopenia Muscle. 2023 Oct 27;14(6):2703–18. doi: 10.1002/jcsm.13346 (PMC10751449; doi:10.1002/jcsm.13346)
Supplement: Supplementary file 3 — Table S2. Primer list. [file JCSM-14-2703-s003.docx]

**Supplementary Table 2. Primer list**

| **Gene** | **Primer** |
| --- | --- |
| *Gipr* | 5’-CCAGAGAAGAATGGGGCTTT-3’(forward)  5’-GTCGTCAGGGACAGGGAGT-3’(reverse) |
| *Pdgfra* | 5’-GACGAGTGTCCTTCGCCAAAGTG-3’(forward)  5’-CAAAATCCGACCAAGCACGAGG-3’(reverse) |
| *Pparg* | 5’-TGGAGACCGCCCAGGCTTG-3’(forward)  5’GTCTGTCATCTTCTGGAGCACCTT-3’(reverse) |
| *Wisp1* | 5’-CAGTGAGCCCAAGAGTCAGG-3’(forward)  5’-TCGTCTCTGTCAGCTTGCAC-3’(reverse) |
| *Bmp3b* | 5’-CTTTGACGCCTACTACTGTGCTG-3’ (forward)  5’-AAGGGAGTTCATCTTGTCTGGAA-3’ (reverse) |
| *Follistatin* | 5’-GCCTGCCACCTGAGAAAGG-3’(forward)  5’-CGCCACACTGGATATCTTCACA-3’(reverse) |
| *18S* | 5’-CTCAACACGGGAAACCTCAC-3’(forward)  5’-CGCTCCACCAACTAAGAACG-3’(reverse) |
